# Dynamics and Entropy Analysis for a New 4-D Hyperchaotic System with Coexisting Hidden Attractors

**DOI:** 10.3390/e21030287

**Published:** 2019-03-15

**Authors:** Licai Liu, Chuanhong Du, Xiefu Zhang, Jian Li, Shuaishuai Shi

**Affiliations:** 1 School of Electronic and Information Engineering, Anshun University, Anshun 561000, China; 2 School of Mathematics and Computer Science, Guizhou Education University, Guiyang 550018, China; 3 School of Information Engineering, Guizhou University of Engineering Science, Bijie 551700, China

**Keywords:** hidden attractor, hyperchaotic system, multistability, entropy analysis

## Abstract

This paper presents a new no-equilibrium 4-D hyperchaotic multistable system with coexisting hidden attractors. One prominent feature is that by varying the system parameter or initial value, the system can generate several nonlinear complex attractors: periodic, quasiperiodic, multiple topology chaotic, and hyperchaotic. The dynamics and complexity of the proposed system were investigated through Lyapunov exponents (LEs), a bifurcation diagram, a Poincaré map, and spectral entropy (SE). The simulation and calculation results show that the proposed multistable system has very rich and complex hidden dynamic characteristics. Additionally, the circuit of the chaotic system is designed to verify the physical realizability of the system. This study provides new insights into uncovering the dynamic characteristics of the coexisting hidden attractors system and provides a new choice for nonlinear control or chaotic secure communication technology.

## 1. Introduction

The chaotic system has great application prospects in the field of image encryption [1,2,3] and secure communication [4]. For a long time, many chaotic systems composed of certain ordinary differential equations have been explored. This produced a series of classic three-dimensional continuous chaotic systems, including the Lorenz [5,6,7], Rossler [8], Chua, Chen [9,10], and Liu systems [11]. By adding linear or nonlinear state feedback controllers on 3-D chaotic systems, various 4-D chaotic systems can be constructed [12,13,14]. Four-dimensional chaotic systems have more complex nonlinear complexity and better randomness than 3-D chaotic systems. These continuous autonomous chaotic systems have common attractors called self-excited attractors because the oscillation is excited from unstable equilibria. At certain initial conditions, traditional self-excited attractors could be tracked from a computational point of view [15].

Recently, the issue of hidden attractors has drawn much attention from the field of nonlinear chaos. The hidden attractors, without equilibrium or with stable equilibrium points, have been found in some continuous chaotic or hyperchaotic systems [16,17]. The basin of attraction for hidden attractors does not intersect with small neighborhoods of any equilibrium point [18,19,20]. Because the system with hidden attractors has neither homoclinic nor heteroclinic orbits, it has completely different dynamic characteristics from the self-excited attractors [21,22,23]. In addition, the coexistence of multiple hidden attractors is a strange physical phenomenon called a multistable system [24,25,26], often encountered in nonlinear dynamic systems. Such a multistable state can greatly improve the complexity of chaotic systems, making these chaotic systems with hidden attractors more suitable for use in chaotic encryption technology. In most cases, however, multistable systems with hidden attractors tend to experience unexpected and potentially disastrous outcomes. Due to the fact that these systems are vulnerable and prone to unpredictably switch to another attractor, multistable systems can cause aircraft crashes [15], drill string failures and breakdowns [27], serious problems during financial crises [28], and catastrophic shifts in ecosystem services [28,29]. Up to now, although predicting a catastrophic bifurcation for multistable systems has been extremely difficult [30], it is still important to uncover and analyze all coexisting attractors in different scientific fields [15].

Motivated by the above research, a new no-equilibrium hyperchaotic system with coexisting hidden attractors is proposed in this work. Up to now, compared with the 3-D hidden attractor system, very little has been published on 4-D hidden attractors, especially for a hyperchaotic system. Therefore, in this paper, a 4-D chaotic system without any equilibrium has been constructed by adding a state variable to a 3-D chaotic system developed by Vaidyanathan and Volos in 2015 [31]. When selecting certain parameters and initial conditions, the Lyapunov exponents (LEs) of the proposed 4-D hyperchaotic system were $LE_{1}=0.0030$, $LE_{2}=-0.0079$, $LE_{3}=0.0044$, and $LE_{4}=0$. There were two positive LEs, and the proposed system had hyperchaotic behavior. At this point, the Kaplan–Yorke fractional dimension $D_{KY}=3.93$.

The existence of chaotic attractors was demonstrated by Lyapunov exponents, a bifurcation diagram, a phase diagram, a time domain diagram, and a power spectral density map. The complexity of the coexisting hidden attractors was also carried out by means of entropy analysis. It was found from the results that the proposed system exhibited extremely complex dynamic characteristics under different initial conditions of the system, such as hidden attractors, quasi-limit cycles, and coexisting attractors with different topological structures. Further, the shape of the hidden attractor was different from the existing attractor. The hidden attractor system was realized by a circuit, for which the experimental results were consistent with the simulation results, further verifying the chaotic characteristics of the system. These qualitative and quantitative studies show that the new system has complex chaotic characteristics.

The rest of this paper is organized as follows. Section 2 describes the mathematical model and chaotic characteristics of the new 4-D system with coexisting hidden attractors. The phenomena exhibited by the system, such as periodicity, multiple coexisting hidden attractors, and quasi-periodic limit cycles, are discussed in Section 3. The information spectral entropy (SE) analysis is given in detail in Section 4. Section 5 presents the circuit of the hyperchaotic system. The inadequacies of the work are discussed in Section 6, and Section 7 summarizes the conclusions.

## 2. System Description

### 2.1. Model of the New Chaotic System

A 3-D conservative no-equilibrium chaotic system was developed by Vaidyanathan and Volos [31]. Vaidyanathan’s system, having LEs of $LE_{1}=-0.0395$, $LE_{2}=0$, and $LE_{3}=-0.0395$ and a Kaplan–Yorke dimension of $D_{KY}=3$, is described as

(1) $$\left\{\begin{array}{l} \dot{x}=ay+xz\\ \dot{y}=-bx+yz\\ \dot{z}=1-x^{2}-y^{2} \end{array}\right.$$

where *x, y,* and *z* are state variables and *a* and *b* are parameters of the system. By adding the fourth state variable *w*, and feeding the third state variable *z* to the fourth variable *w*, a new 4-D hyperchaotic system is obtained as

(2) $$\left\{\begin{array}{l} \dot{x}=ay+xz\\ \dot{y}=-bx+yz\\ \dot{z}=1-x^{2}-y^{2}\\ \dot{\omega }=z(\omega -1) \end{array}\right.$$

where *x, y, z,* and *w* are state variables and *a* and *b* are positive real constant parameters of the system. We know that the equilibrium points of system (2) can be achieved by solving the roots of Equation (3). Simplifying and reorganizing Equation (3), we could get $ay^{2}+bx^{2}=0$. Considering that $1-x^{2}-y^{2}=0$ and *a* and *b* are positive real numbers, Equation (3) has no solution; that is, there is no equilibrium point. According to the definition of hidden attractors, the system’s attractors belong to hidden attractors. Its basin of attraction does not contain neighborhoods of equilibria [32,33].

(3) $$\left\{\begin{array}{l} ay+xz=0\\ -bx+yz=0\\ 1-x^{2}-y^{2}=0\\ z(\omega -1)=0 \end{array}\right.$$

### 2.2. Nonlinear Description of the System

**Figure 1:**
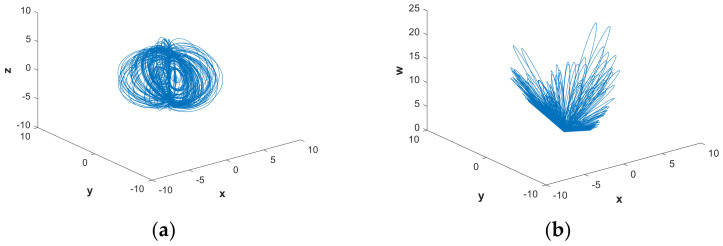

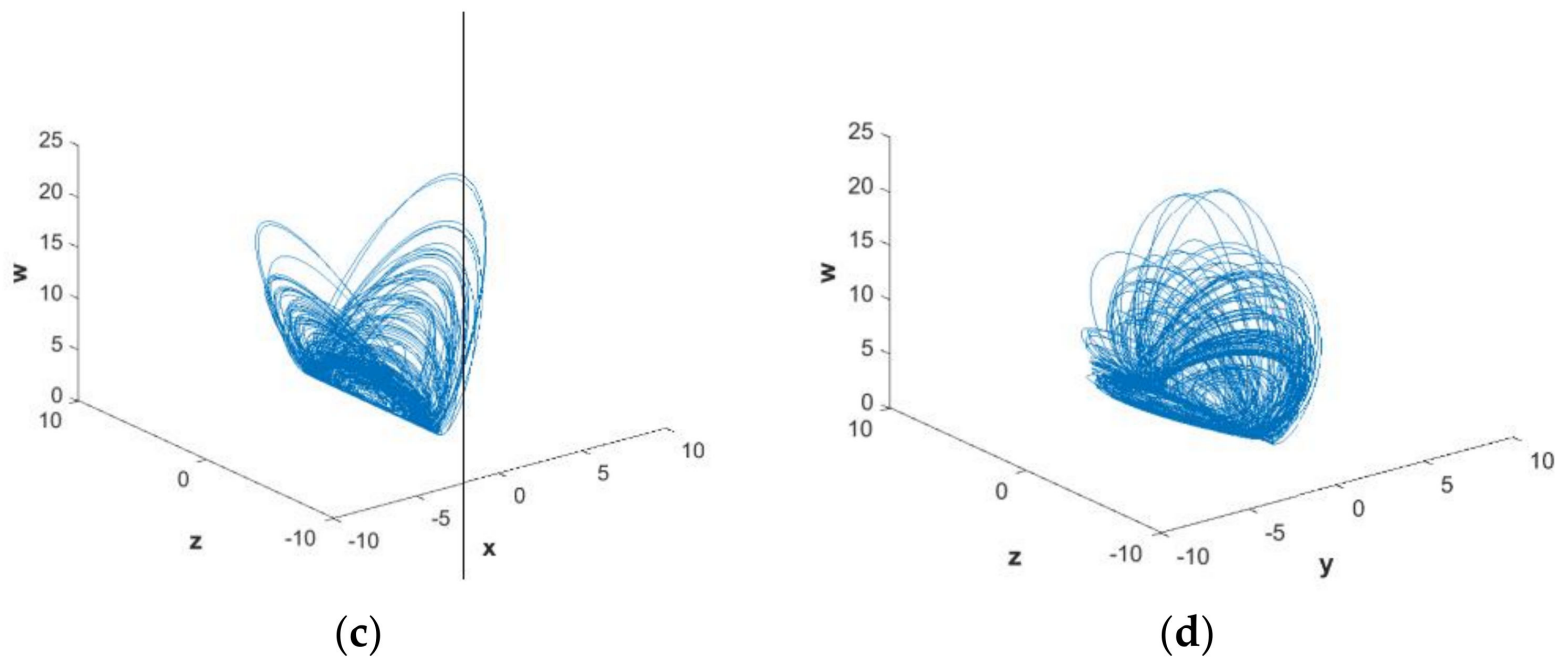
Three-dimensional chaotic attractor of system (2) with parameters a=0.05, b=1 and initial conditions $\left(x_{0},y_{0},z_{0},w_{0})=(-1,-1,4,4\right)$ on the (**a**) (x,y,z) space, (**b**) (x,y,w) space, (**c**) (x,z,w) space, and (**d**) (y,z,w) space.

**Figure 2:**
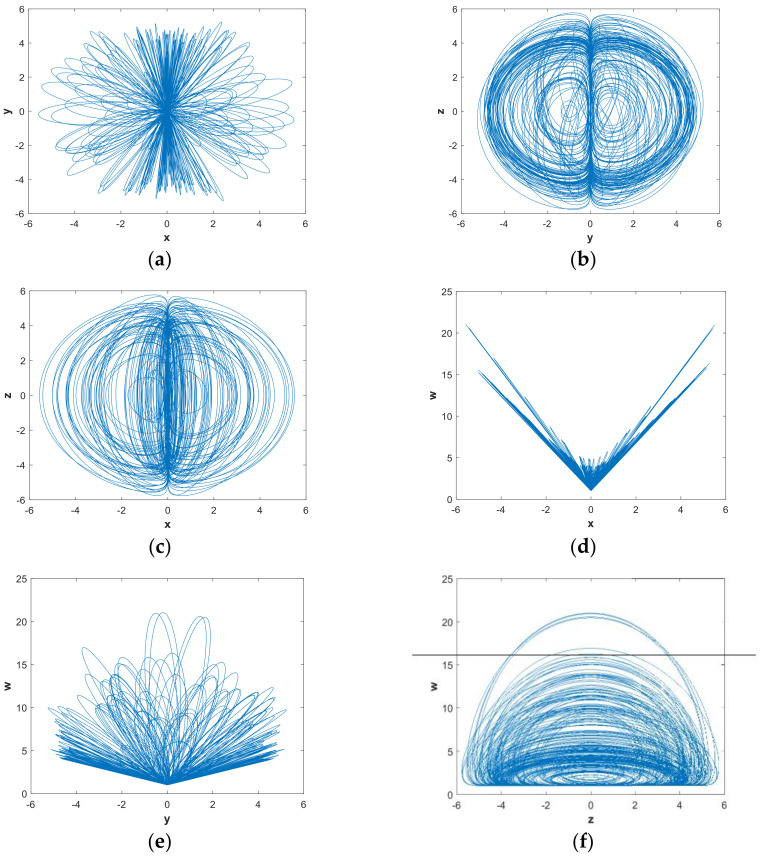
Two-dimensional chaotic attractor of system (2) with parameters a=0.05, b=1 and initial conditions $\left(x_{0},y_{0},z_{0},w_{0})=(-1,-1,4,4\right)$: (**a**) x−y plane; (**b**) y−z plane; (**c**) x−z plane; (**d**) x−w plane; (**e**) y−w plane; and (**f**) z−w plane.

**Figure 3:**
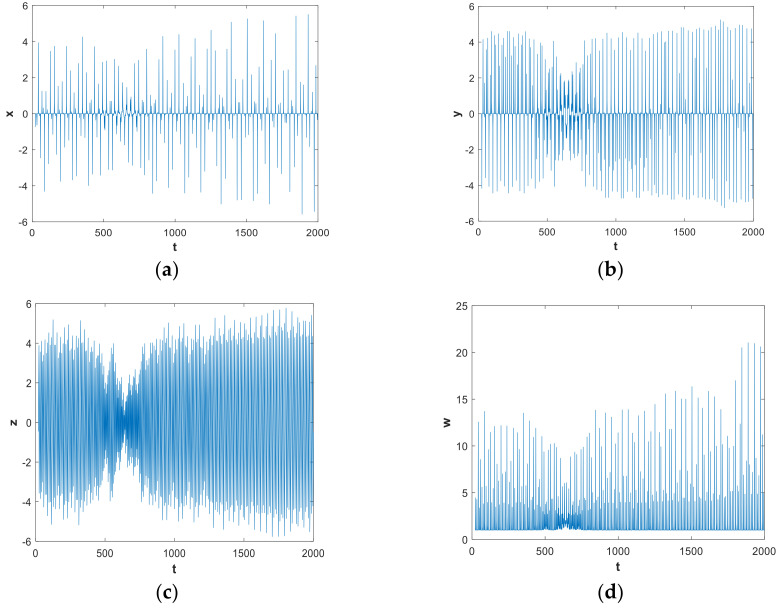
Time series of system (2) with parameters a=0.05, b=1 and initial conditions $\left(x_{0},y_{0},z_{0},w_{0})=(-1,-1,4,4\right)$: (**a**) x variable; (**b**) y variable; (**c**) z variable; and (**d**) w variable.

**Figure 4:**
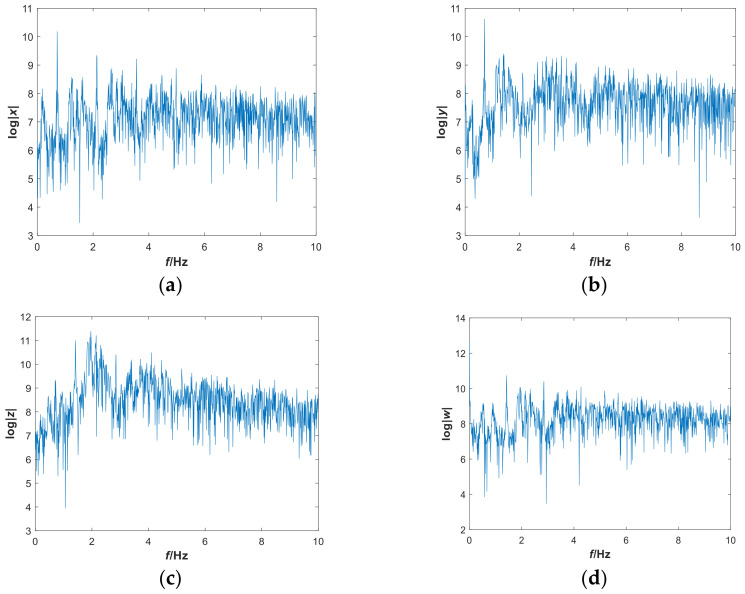
Frequency spectrum of system (2) with parameters a=0.05, b=1 and initial conditions $\left(x_{0},y_{0},z_{0},w_{0})=(-1,-1,4,4\right)$: (**a**) the x variable; (**b**) y variable; (**c**) z variable; and (**d**) w variable.

In this subsection, we mainly discuss the nonlinear dynamics of the system with hidden attractors by means of numerical simulation. If there is no special explanation, the simulation step size is 0.01, the ode45 numerical solver is used, and the simulation time is 2000 s in this paper. Figure 1 shows the 3-D attractor projection of system (2), and Figure 2 depicts the 2-D attractor projection of system (2). From the projection phase diagram it can be found that the attractors are different from the scroll or wing shape and belong to a new attractor. Compared with the attractors of system (1), the new system has more abundant attractors, so the dynamic characteristics of the new system are more complicated. Figure 3 is a time series diagram of four state variables of system (2) that indicates that the system is aperiodic, which corresponds with the chaotic feature. The curves in Figure 4, obtained by Fourier transform of the autocorrelation function, show that the variables are continuous and there are no obvious peaks, which accords with the characteristics of chaos.

**Figure 5:**
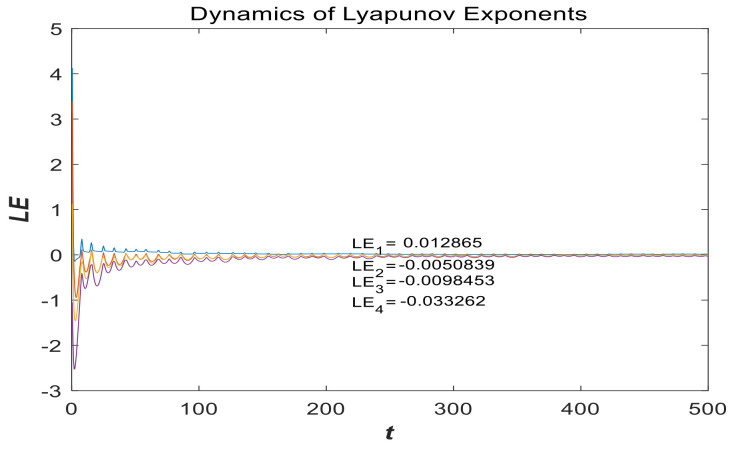
LEs of system (2) in dependence on parameters a=0.05, b=1 and initial value $\left(x_{0},y_{0},z_{0},w_{0})=(-1,-1,4,4\right)$.

Using the parameters a=0.05, b=1 and initial conditions $\left(x_{0},y_{0},z_{0},w_{0})=(-1,-1,4,4\right)$, Figure 5 illustrates the LEs of the system computed with the Wolf algorithm [34,35]. They are $LE_{1}=0.012865$, $LE_{2}=-0.0050839$, $LE_{3}=-0.0098453$, and $LE_{4}=-0.033262$, respectively. One of them is positive and two of them are negative, and the sum of the LEs is negative, so the system is a stable chaotic system with hidden attractors. A further discussion of LEs is given in Section 3.2, which demonstrates that the system is hyperchaotic under certain parameters and proper initial conditions.

**Figure 6:**
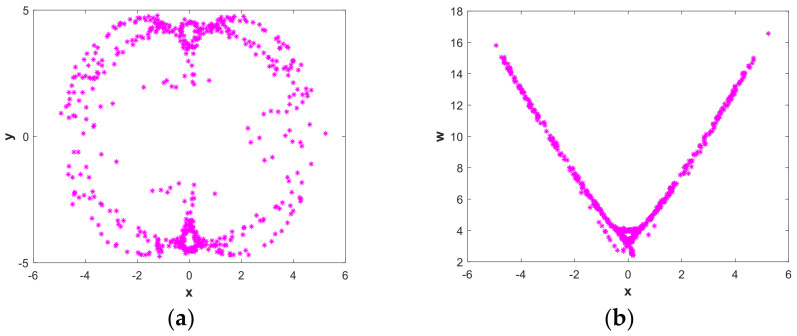
Poincaré map of system (2) in dependence on parameters a=0.05, b=1 and initial value $\left(x_{0},y_{0},z_{0},w_{0})=(-1,-1,4,4\right)$ in the (**a**) x−y plane and (**b**) x−ω plane.

To further illustrate that system (2) is chaotic, Figure 6 illustrates a Poincaré map [36,37,38] with a=0.05, b=1 and initial value $\left(x_{0},y_{0},z_{0},w_{0})=(-1,-1,4,4\right)$. Figure 6(a) and (b) are Poincaré maps in the x−y and x−w planes on a z=0 cross section. As is shown in the cross section of the map, there is a set of points distributed along the line or curve arc with a self-similar fractal structure. Therefore, the Poincaré map of the system also shows the properties of chaos.

## 3. Nonlinear Dynamics of the System

To date, knowledge of hidden attractors is still insufficient and research results are in their early stages. There is a great need to address the characteristics of nonlinear system with hidden attractors. What we present in this section is an analysis of the chaotic, hyperchaotic, and multistable characteristics of the proposed system in terms of system parameters and initial values of state variables. More specifically, the phenomena of periodicity, coexistence of multiple hidden attractors, and quasi-periodic limit cycles are analyzed and explained in great detail.

### 3.1. Influence of Parameters on System Dynamic Characteristics

A bifurcation diagram can show the relationship between a system and the variation of parameters and changes in dynamic behaviors, as well as graphically reflect nonlinear behaviors such as chaotic, periodic, and quasi-periodic limit cycles in the system. Therefore, in this section, a bifurcation diagram is used to analyze the influence of different system parameters on the dynamic characteristics of system (2).

**Figure 7:**
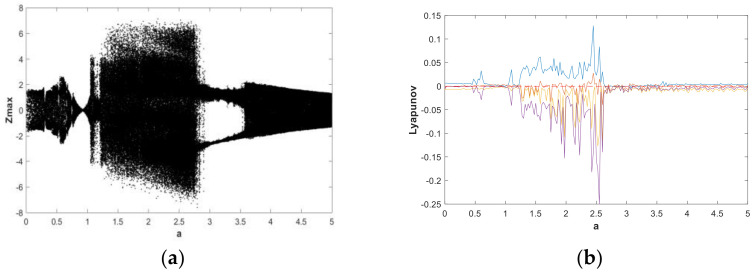
Bifurcation diagram and LEs of system (2) about a, with b=0.05, initial value $\left(x_{0},y_{0},z_{0},w_{0})=(2,0,0,0\right)$, and a∈(0,5): (**a**) bifurcation diagram; (**b**) LE graphs.

**Figure 8:**
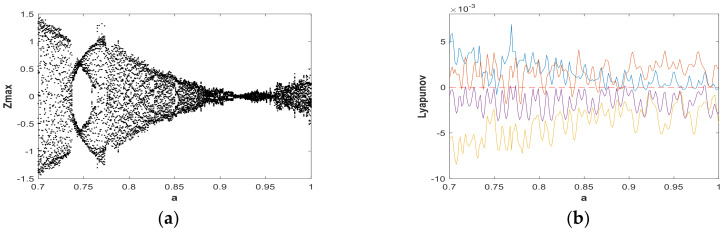
Bifurcation diagram and LEs of system (2) about a, with b=0.05, initial value $\left(x_{0},y_{0},z_{0},w_{0})=(2,0,0,0\right)$, and a∈(0.7,1): (**a**) bifurcation diagram; (**b**) LE graphs.

**Figure 9:**
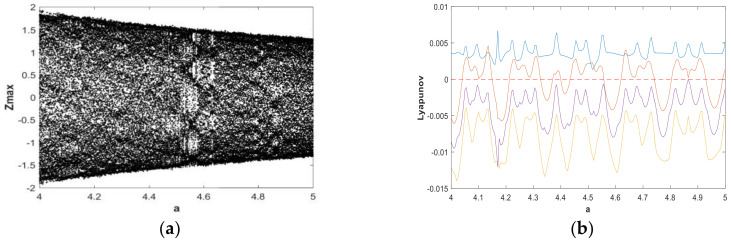
Bifurcation diagram and LEs of system (2) about a, with b=0.05, initial value $\left(x_{0},y_{0},z_{0},w_{0})=(2,0,0,0\right)$, and a∈(4,5): (**a**) bifurcation diagram; (**b**) LE graphs.

The change of dynamic behaviors is shown in the bifurcation diagram in Figure 7(a), from which it can be seen that when fixing b=0.05 and a∈(0,5) with the initial value $\left(x_{0},y_{0},z_{0},w_{0})=(2,0,0,0\right)$, the transition to chaos was apparent and the system was in a wide-domain chaotic state when the value of *a* increased. When a∈(1.2,2.7), it was obvious that the system was in a chaotic state. Figure 7(b) is the corresponding LE diagram of the system under this simulation parameter. Figure 7(b) shows that two positive Lyapunov exponents also appeared in some ranges, indicating hyperchaos in system (2). When a∈(2.7,3.6), the maximum Lyapunov exponent of the system was very close to 0, while the remaining LEs were negative or zero, indicating that the system was in the limit circle state or had a hidden periodic attractor. In order to facilitate the analysis of the dynamic behavior, the bifurcation graph and corresponding LE graph when a∈(0.7,1) and a∈(4,5) were enlarged locally, as shown in Figure 8 and Figure 9, respectively. Duringa∈(0.7,1) and a∈(4,5), Figure 8 and Figure 9 show that there were complex nonlinear behaviors in these two regions. In addition, the bifurcation boundary line had a certain width of point set, as opposed to being a single point line, which made the system have more abundant dynamic behaviors. Further, because the LE graphs show that the system had two positive LEs, hyperchaotic behavior existed in a large range.

**Figure 10:**
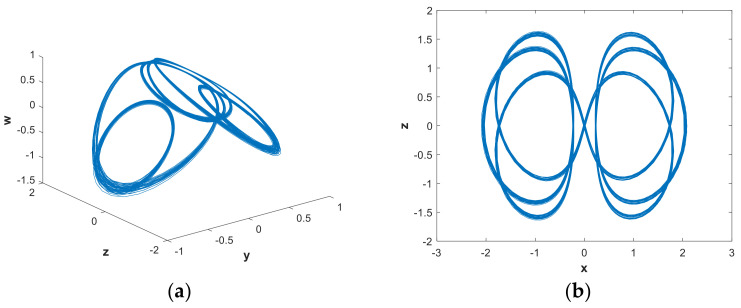
Projections of hidden attractors with parameters a=0.88922, b=0.05 and initial value $\left(x_{0},y_{0},z_{0},w_{0})=(2,0,0,0\right)$: (**a**) attractor in the y−z−w space; (**b**) attractor in the x−z plane.

**Figure 11:**
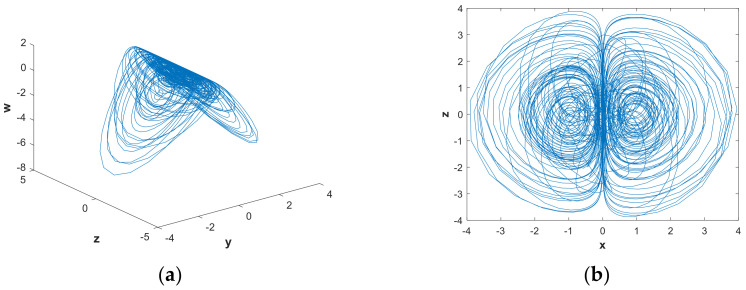
Projections of hidden attractors with parameters a=1.0621, b=0.05 and initial value $\left(x_{0},y_{0},z_{0},w_{0})=(2,0,0,0\right)$: (**a**) attractor in the y−z−w space; (**b**) attractor in the x−z plane.

**Figure 12:**
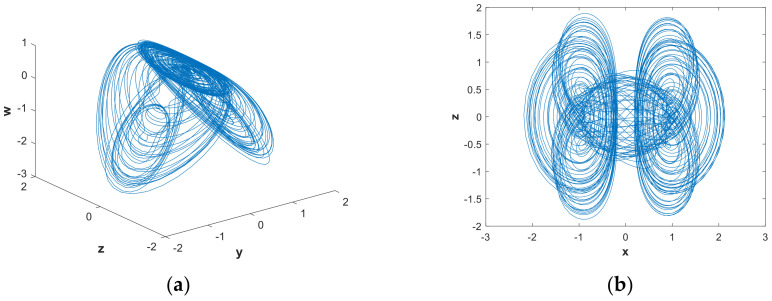
Projections of hidden attractors with parameters a=1.2, b=0.05 and initial value $\left(x_{0},y_{0},z_{0},w_{0})=(2,0,0,0\right)$: (**a**) attractor in the y−z−w space; (**b**) attractor in the x−z plane.

**Figure 13:**
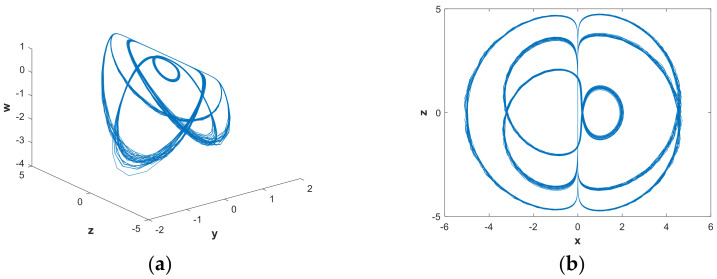
Projections of hidden attractors with parameters a=2.982, b=0.05 and initial value $\left(x_{0},y_{0},z_{0},w_{0})=(2,0,0,0\right)$: (**a**) attractor in the y−z−w space; (**b**) attractor in the x−z plane.

**Figure 14:**
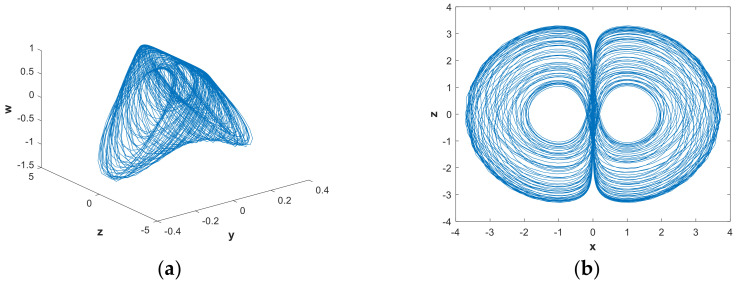
Projections of hidden attractors with parameters a=4.9, b=0.05 and initial value $\left(x_{0},y_{0},z_{0},w_{0})=(2,0,0,0\right)$: (**a**) attractor in the y−z−w space; (**b**) attractor in the x−z plane.

In order to further analyze the influence of parameters on the hidden attractors system, the system was analyzed using a phase diagram. When a=0.88922, Figure 8(b) shows that the system had a positive LE close to 0 and three negative LEs. The system was in a state of quasi-periodic limit cycle. The 3-D and 2-D projections of the hidden attractor diagram of the system are shown in Figure 10. By increasing the value of parametera, when a=1.0621, a hidden attractor could be observed (Figure 11). When a=1.2, the LEs were $LE_{1}=0.0030$, $LE_{2}=-0.0079$, $LE_{3}=0.0044$, and $LE_{4}=0$, so the system was hyperchaotic. From Figure 12, we can see that there were many strange attractors with different topologies. When a=2.982, the phase diagram of the system was a quasi-periodic limit cycle, as shown in Figure 13, which is consistent with the illustration in Figure 7. It can be seen from Figure 9 that when a∈(4,5), the bifurcation phenomenon was complicated; hence, the system dynamics behavior was also rich. Figure 9(b) shows that when a=4.9, the system was in a hyperchaotic state, having hidden attractors, as shown in Figure 14. In this scenario, the hidden attractors in the system were different from the attractors when the other parameters were taken. They were novel and unique hidden attractors.

According to the above analysis, there are many kinds of hidden attractors and different topologies in system (2). Therefore, this system has novel attractors in a variety of shapes and has rich dynamic behavior.

### 3.2. Influence of Initials on System Dynamic Characteristics

More recently, the impact of initial values on the dynamic behavior of a system with hidden attractors has been subject to considerable discussion [17,39,40,41]. Here, we focus on the influence of different initial values on the dynamic characteristics of the hidden attractors in terms of a phase diagram (i.e., projections of attractor) and a bifurcation diagram.

**Figure 15:**
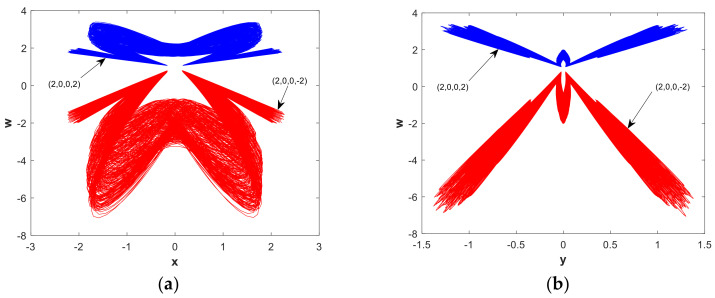
Projections of hidden attractors with different initial conditions. Blue attractors‘ initial is (2,0,0,2), red attractors‘ initial is(2,0,0,−2), and the parameters are a=1, b=0.05: (**a**) attractor in the x−w plane; (**b**) attractor in the y−w plane.

With the parameters of system (2) chosen as a=1, b=0.05, Figure 15 shows the phase diagram of the hidden attractors. In Figure 15, the blue attractors’ initial is (2,0,0,2), and the red attractors’ initial is$\left(x_{0},y_{0},z_{0},w_{0})=(2,0,0,-2\right)$. It can be seen from the phase diagram that the system space corresponding to these two initial values had a certain symmetrical similarity. Specifically, the size was different and the phase was opposite.

In order to facilitate the analysis of the influence of different system initial conditions on system dynamics under the same system parameters, the bifurcation diagram and LEs were used again.

**Figure 16:**
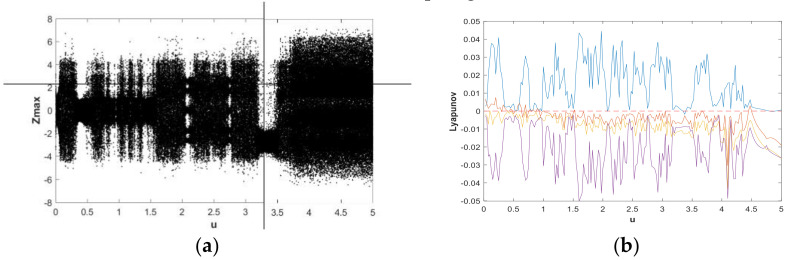
Bifurcation diagram and LEs of system (2) with a=1, b=0.05, initial value Y0=(u,u,0,0), and u∈[0,5]: (**a**) bifurcation diagram; (**b**) LE graphs.

**Figure 17:**
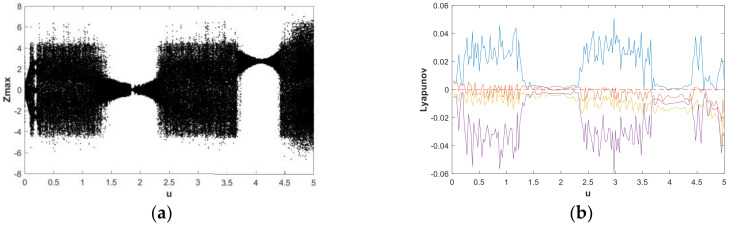
Bifurcation diagram and LEs of system (2) with a=1, b=0.05, initial value Y1=(u,0,0,0), and u∈[0,5]: (**a**) bifurcation diagram; (**b**) LE graphs.

**Figure 18:**
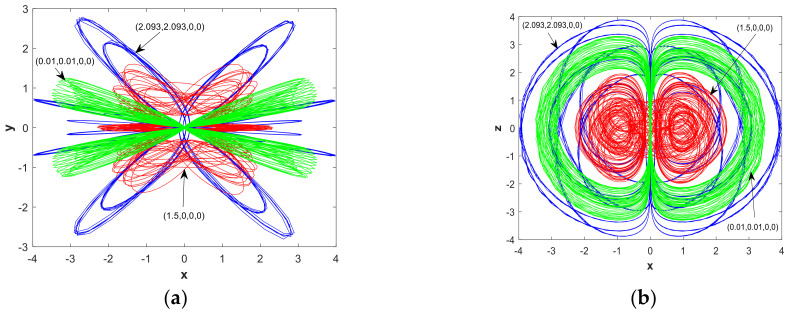

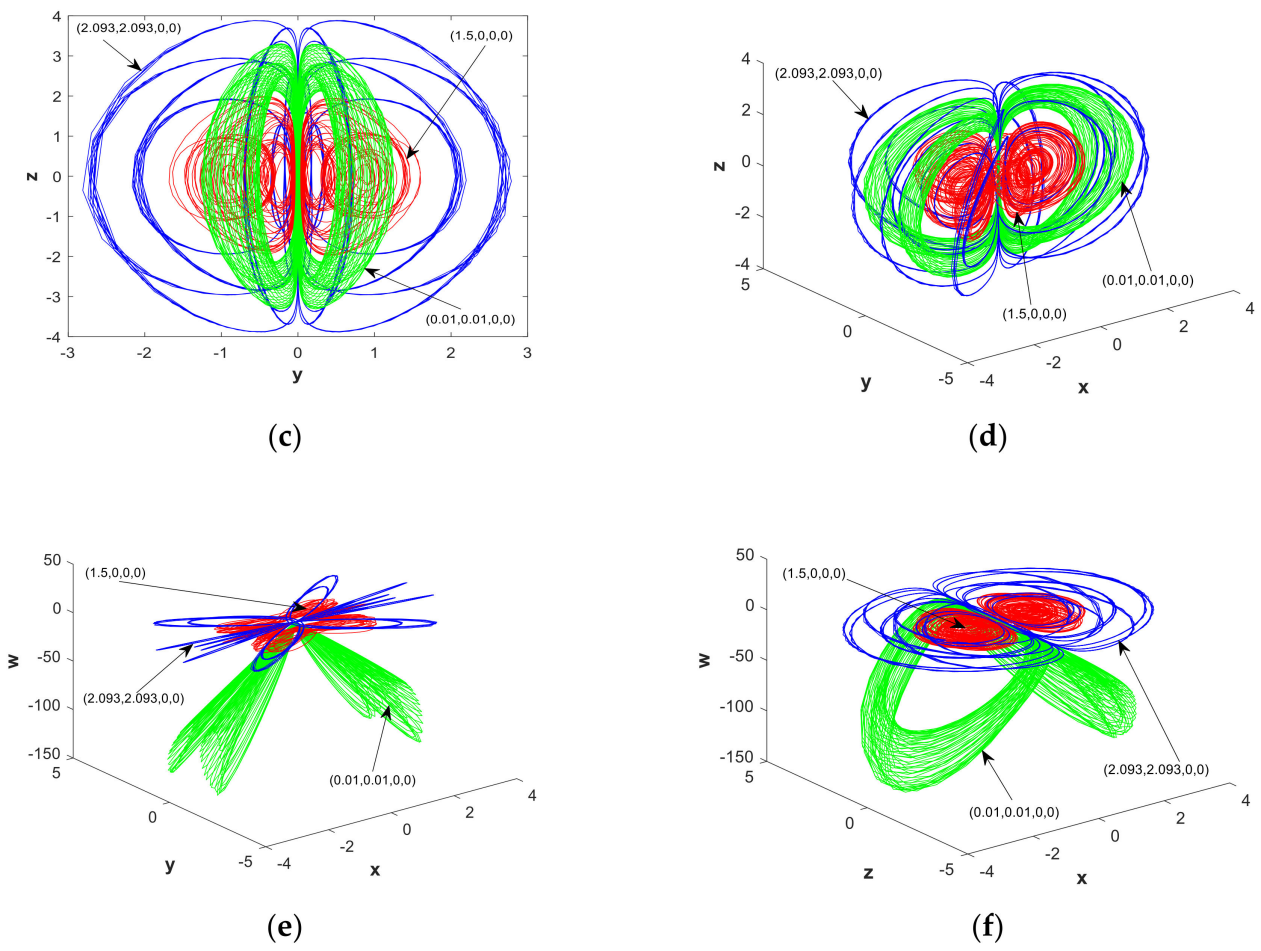
Projections of hidden attractors with different initial conditions. Green attractors‘ initial is (0.01,0.01,0,0), blue attractors‘ initial is (2.093,2.093,0,0), red attractors‘ initial is(1.5,0,0,0), and parameters a=1, b=0.05: (**a**) attractor in the x−y plane; (**b**) attractor in the x−z plane; (**c**) attractor y−z plane; (**d**) in the x−y−z space; (**e**) attractor in the x−y−w space; and (**f**) attractor in the x−z−w space.

There were two initial values of the system Y0=(u,u,0,0) and Y1=(u,0,0,0), respectively andu∈[0,5]. The parameters chosen were a=1, b=0.05. Figure 16 and Figure 17 present bifurcation diagrams and LEs of system (2) when increasing the value of *u*. The bifurcation diagrams and LEs shown in Figure 16 and Figure 17 exhibit the process of periodic limit cycles, quasi-periodic limit cycles, and hyperchaos under different initial values. The corresponding LE graphs are also consistent with the bifurcation diagram change. With the Y0 point, let u=0.01 and u=2.093, respectively, and for the Y1 point, let u=1.5. Thus, we have chosen the initial values (0.01,0.01,0,0),(2.093,2.093,0,0), and(1.5,0,0,0). The colors corresponding to these three initial values are green, blue, and red, respectively. The 2-D numerical simulation phase diagram is shown in Figure 18. Combining Figure 16 and Figure 17, it can be found from the phase diagram of Figure 18 that when the initial value was (0.01,0.01,0,0), the system was in a weak chaotic state; when the initial value was (2.093,2.093,0,0), $LE_{1}=0$, $LE_{2}=-0.0076601$, $LE_{3}=-0.0067299$, and $LE_{4}=-0.0075862$, the system was in a quasi-periodic limit cycle; and when the initial value was (1.5,0,0,0), $LE_{1}=0.0029082$, $LE_{2}=-0.0034458$, $LE_{3}=0$, and $LE_{4}=-0.0017509$, the system was in a hyperchaotic state. It was also found that the chaotic systems corresponding to different initial values contained hidden attractors with different topological structures.

Based on the above analysis, it can be concluded that the system is multistable and can produce complex multiple coexisting hidden attractors.

## 4. Information of Spectral Entropy Analysis

In order to measure the dynamic complexity of chaotic systems (2) with strange hidden attractors, in this section, we discuss system complexity by means of information spectral entropy (SE) analysis [42,43].

We know that another statistical property of dynamical systems is SE, which has a certain relationship with LEs and the Hausdorff dimension. SE is a measure of the chaotic properties of the system. The greater the system complexity value, the stronger the randomness of the system. When such a system is used as a communication key, the security of the information is higher. The complexity of chaotic systems is generally divided into behavioral complexity and structural complexity. At present, there are several algorithms for calculating the complexity of chaotic system behavior, and they are based on the Kolmogorov method and Shannon’s entropy. These algorithms are fast and have accurate results. However, the calculation results of high-dimensional chaotic systems will overflow, which may result in the expected results. The structural complexity is the analysis of the energy characteristics in the transform domain. The scope of its action is the entire sequence of the system, not locally, so the results are more global than the behavioral complexity algorithm [18]. In this paper, the SE algorithm of structural complexity was used to analyze the dynamic characteristics of the system.

### 4.1. Spectral Entropy Complexity Algorithm

By using Fourier transform, the energy distribution was obtained. After that, the corresponding SE value was obtained by combining Shannon entropy. The algorithm requires the following steps.

For the chaotic pseudorandom sequence $\left\{x(n),n=0,1,2,3,\cdots ,N-1\right\}$ of length N, the DC part is removed by Equation (4) so that the spectrum more effectively reflects the energy information of the signal:

(4) $$x(n)=x(n)-\bar{x}$$

where $\bar{x}=\frac{1}{N}\sum_{n=0}^{N-1}x(n)$. A discrete Fourier transform (DFT) was then performed on the x(n) to obtain Equation (5):

(5) $$X(k)=\sum_{n=0}^{N-1}x(n)e^{-j\frac{2\pi }{N}nk}=\sum_{n=0}^{N-1}x(n)W_{N}{}^{nk}$$

where k=0,1,2,3,⋯,N−1. The relative power spectrum was calculated for the transformed sequence X(k) by taking the first half of the sequence for calculation. According to the Parseval theorem, the power spectrum value of a certain frequency point is determined by Equation (6):

(6) $$S(k)=\frac{{\left|X(k)\right|}^{2}}{N}$$

where $k=0,1,2,3,\cdots ,\frac{N}{2}-1$. Then, the total power of the sequence can be defined as Equation (7):

(7) $$S_{total}=\frac{\sum_{k=0}^{N/2-1}{\left|X(k)\right|}^{2}}{N}$$

The probability of the relative power spectrum for the sequence is shown in Equation (8):

(8) $$p_{k}=\frac{S(k)}{S_{total}}=\frac{\frac{1}{N}{\left|X(k)\right|}^{2}}{\frac{1}{N}\sum_{k=0}^{N/2-1}{\left|X(k)\right|}^{2}}=\frac{{\left|X(k)\right|}^{2}}{\sum_{k=0}^{N/2-1}{\left|X(k)\right|}^{2}}$$

From statistical knowledge, we know that $\sum_{k=0}^{N/2-1}p_{k}=1$. Combined with the Shannon entropy solving method, the SE of the signal could be obtained using Equation (9):

(9) $$SE=\sum_{k=0}^{N/2-1}p_{k}\ln\frac{1}{p_{k}}$$

In Equation (9), if $p_{k}=0$, then $p_{k}\ln p_{k}=0$. The equation converges to $\ln(\frac{N}{2})$, and for the convenience of comparative analysis, the SE is normalized. Then, the normalized SE calculation formula could be obtained with Equation (10) [42]:

(10) $$SE(N)=\frac{SE}{\ln(N/2)}$$

It can be seen from the above algorithm process that the higher the degree of imbalance for the sequence power spectrum distribution, the simpler the structure of the sequence spectrum, resulting in a stronger law of oscillation for the signal. Correspondingly, a smaller SE stands for smaller complexity; otherwise, the complexity is greater.

### 4.2. Influence of Parameters on Entropy

**Figure 19:**
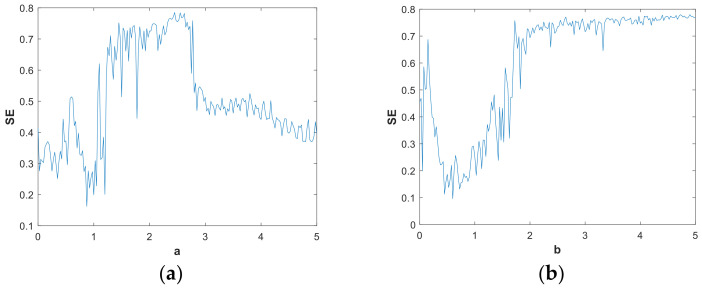
SE vs. parameters of the system, initial is (x,y,z,ω)=(2,0,0,0): (**a**) b=1, a∈(0,5); (**b**) a=0.05, b∈(0,5).

It can be seen from the discussion in Section 3.1 that the change of system parameters has a great influence on the nonlinear dynamic behavior of the system, which affects the complexity of the system. Therefore, studying the influence of system parameters on SE is necessary. Figure 19 shows how parameters *a* and *b* impact the SE, where parameter a=1 or parameter b=0.05, and the initial (x,y,z,ω)=(2,0,0,0). With the great fluctuations around a=1, the SE attenuated to 0.1. This is because the system at this time was in the quasi-periodic limit-cycle state with minimal complexity, as shown in Figure 10. Figure 19 indicates that when SE∈(0,0.5), the regions of parameter *a* were greater than parameter *b*. So, in this region, parameter *b* was more sensitive than parameter *a* in terms of change. In the range of b∈(2,5), the SE value was high, and the fluctuation was not large. It is worth noting that when a,b∈(2,2.8), there was a similar complexity value.

### 4.3. Influence of Initials on Entropy

**Figure 20:**
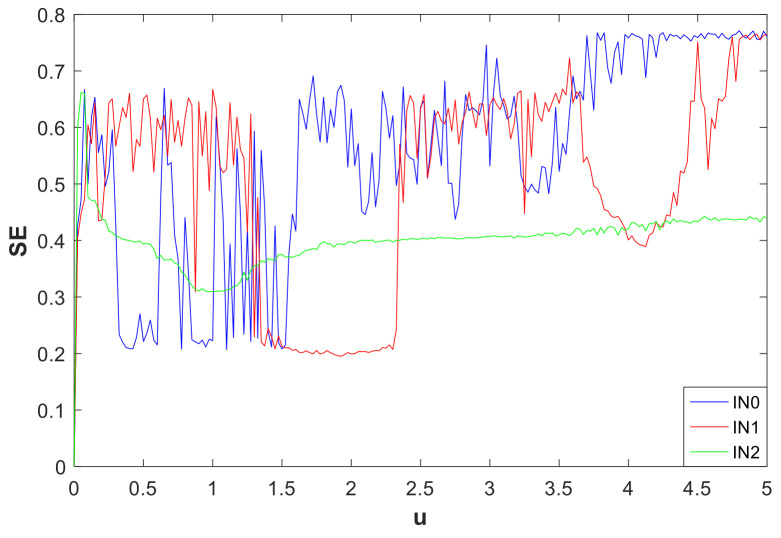
SE vs. initials of the system, whereIN0=(u,u,0,0) (Blue), IN1=(u,0,0,0) (Red), and IN2=(0.01u,0,0,−2u) (Green); u∈(0,5).

The initial value condition is a major factor affecting the dynamic behavior of the system, which was introduced in Section 3.2. In order to study the influence of the initial value of the system on the nonlinear behavior of the system, the degree of influence of the initial value on the nonlinear behavior of the system was further measured from the perspective of entropy. In the SE graphs of Figure 20, we used the parameters a=1, b=0.05. Suppose there are three types of initial conditions: IN0=(u,u,0,0), IN1=(u,0,0,0), and IN2=(0.01u,0,0,−2u), respectively; *u* is a variable, and u∈(0,5). The relationship between the variables *u* and SE is shown in Figure 20. As can be seen from the figure, for the system complexity, the SE of IN0, IN1, and IN2 were alternate variations when u∈(0,0.3022); when u∈(0.3022,0.6179), the SE was IN1>IN2>IN0; when u∈(0.6179,0.83), IN1 was always greater than the IN2 and IN0; when u∈(0.83,1.0076), IN1>IN2>IN0; when u∈(1.0076,1.5503), IN1 was in the transition from chaotic to nonchaotic; when u∈(1.5503,2.3397), the SE was IN0>IN2>IN1; the values of IN1 and IN0 alternated but were always greater than the IN2 when u∈(2.3397,3.985); when u∈(3.985,4.2637), it was approximately IN0>IN2>IN1; when u∈(4.2637,5), the value of SE was IN0>IN1>IN2. As is shown in Figure 20, the change of IN2 was relatively flat, the corresponding system had less complexity, and the dynamic behavior of the system was much less than that of the other two initial value systems. The IN0 curve fluctuations were varied and intense, so the corresponding system was rich in dynamic behavior. The IN1 curve changes were more orderly, and the corresponding system also contained rich nonlinear characteristics.

The above analysis only deals with three simple forms of initial value problems. Since there are numerous initial values for the system, the complexity of the system varies with the initial value and corresponds to infinite variety, which further illustrates that the system has a very rich dynamic behavior.

### 4.4. Characteristic Analysis of Chaotic Diagram of System Entropy

**Figure 21:**
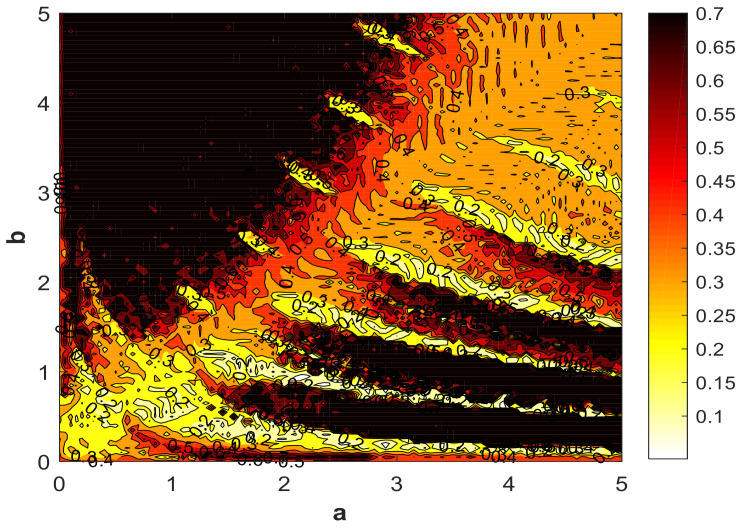
Chaotic characteristics of SE vs. the parameters of the system, with a∈(0,5), b∈(0,5), and the initial $\left(x_{0},y_{0},z_{0},w_{0})=(2,0,0,0\right)$.

The previous sections described the relationship between system parameters, initial system values, and system nonlinear dynamics complexity. In the following section, the chaotic characteristic distribution of the system complexity is discussed from the perspective of the interaction of system parameters *a* and *b*. In order to observe the distribution of chaotic SE more clearly and graphically, a contour map with different color schemes is used to show the chaotic property of SE, using flipud (hot) mode. A contour map was obtained of the complexity of the chaotic system vs. system parameters, as shown in Figure 21, where a∈(0,5), b∈(0,5), and the initial $\left(x_{0},y_{0},z_{0},w_{0})=(2,0,0,0\right)$. The figure shows that under the same initial value, the adjacent different color boundary lines are the contour lines; that is, the adjacent contour lines are filled with the same color, and the color used for filling was done by flipud (hot) mode. Due to the variety of colors in the figure, there are many values for the corresponding system entropy, which is consistent with the results discussed in Section 4.2. The main color distribution in the graph is red and black, or similar, and the corresponding system has a larger entropy value, whereas the other colored areas are smaller. This shows that the system was in a hyperchaotic or chaotic state in a wide range when varying parameters *a* or *b*, which is consistent with the conclusion from the system bifurcation diagram. Therefore, a more detailed color distribution contour map can be obtained by way of subdividing system parameters and changing the system initial value.

## 5. Circuit Design

The nonlinear dynamic behavior of system (2) is discussed here in detail based on numerical simulation, which verified that the system had abundant dynamic behavior. This section presents a novel circuit implementation for a hidden attractor system.

### 5.1. Improved Modular Circuit Design

Electronic circuit realization is of great physical significance for the application of chaos theory [14,44]. Chaotic circuit design mainly includes three methods: individualized design, modular design, and improved modular design. Although an individualized design needs fewer circuit components, it does require more prior knowledge. The modular design approach is based on a dimensionless state equation that is universal and versatile but requires more components. The improved modular design method can combine the advantages of the former two methods very well. By comparing the state differential equation with the actual circuit’s state differential equation, the total coefficients of the system can be determined, which can minimize the number of components. Therefore, we adopted the improved modular design method to design the system circuit.

We used TL082 operational amplifiers, AD633 analog multipliers, some linear resistors, and capacitors to form this 4-D signal generator circuit system. Since the power supply voltage was ±15V, in order to make the signal of the system not exceed the linear dynamic range of the operational amplifier with ±13.5V, the state variable was rescaled using Equation (11). Introduce new state variables $u_{x}$, $u_{y}$, $u_{z}$, and $u_{w}$. Let

(11) $$\left\{\begin{array}{l} x=u_{x}\\ y=u_{y}\\ z=u_{z}\\ w=2u_{w} \end{array}\right.$$

Substituting Equation (11) in Equation (2), we obtain Equation (12):

(12) $$\left\{\begin{array}{l} \frac{du_{x}}{dt}=au_{y}+u_{x}u_{z}\\ \frac{du_{y}}{dt}=-bu_{x}+u_{y}u_{z}\\ \frac{du_{z}}{dt}=1-u_{x}{}^{2}-u_{y}{}^{2}\\ \frac{du_{w}}{dt}=0.5u_{z}(2u_{w}-1) \end{array}\right.$$

In order to make the circuit parameters better match the system, set the time scale to $\tau_{0}$, and $\tau_{0}=10^{4}$; then, perform time transformation. The new time variable is τ, and $t=\tau_{0}\tau$; then, $dt=\tau_{0}d\tau$. Hence, Equation (12) can be written as

(13) $$\left\{\begin{array}{l} \frac{du_{x}}{d\tau }=\tau_{0}(au_{y}+u_{x}u_{z})\\ \frac{du_{y}}{d\tau }=\tau_{0}(-bu_{x}+u_{y}u_{z})\\ \frac{du_{z}}{d\tau }=\tau_{0}(1-u_{x}{}^{2}-u_{y}{}^{2})\\ \frac{du_{w}}{d\tau }=0.5\tau_{0}u_{z}(2u_{w}-1) \end{array}\right.$$

From the constraint relationship of Equation (13), the corresponding circuit equation can be designed as (14):

(14) $$\left\{\begin{array}{l} \frac{du_{x}}{d\tau }=\frac{1}{C_{1}}(\frac{u_{y}}{R_{1}}+\frac{g_{1}u_{x}u_{z}}{R_{2}})\\ \frac{du_{y}}{d\tau }=\frac{1}{C_{2}}(-\frac{u_{x}}{R_{3}}+\frac{g_{2}u_{y}u_{z}}{R_{4}})\\ \frac{du_{z}}{d\tau }=\frac{1}{C_{3}}(\frac{1}{R_{7}}-\frac{g_{3}u_{x}{}^{2}}{R_{5}}-\frac{g_{4}u_{y}{}^{2}}{R_{6}})\\ \frac{du_{w}}{d\tau }=\frac{1}{C_{4}}(\frac{g_{5}u_{w}}{R_{9}}-\frac{1}{R_{8}})u_{z} \end{array}\right.$$

where $C_{1}$, $C_{2}$, $C_{3}$, and $C_{4}$ are the integral capacitance; $g_{1}$, $g_{2}$, $g_{3}$, and $g_{4}$ are the gain of five multipliers; $u_{x}$, $u_{y}$, $u_{z}$, and $u_{w}$ are the output variables of the integrator, which correspond to the system-state variable of Equation (13); and $R_{i}$(i=1,2,3,⋯9) is the corresponding resistance. In order to get the parameters of the circuit, we compared Equations (13) and (14), and then obtained Equation (15). Thus, the specific resistance value is shown in Equation (16):

(15) $$\left\{\begin{array}{l} \frac{1}{C_{1}R_{1}}=a\tau_{0}\\ \frac{g_{1}}{C_{1}R_{2}}=\tau_{0}\\ \frac{1}{C_{2}R_{3}}=b\tau_{0}\\ \frac{g_{2}}{C_{2}R_{4}}=\tau_{0}\\ \frac{g_{3}}{C_{3}R_{5}}=\tau_{0}\\ \frac{g_{4}}{C_{3}R_{6}}=\tau_{0}\\ \frac{1}{C_{3}R_{7}}=\tau_{0}\\ \frac{1}{C_{4}R_{8}}=0.5\tau_{0}\\ \frac{g_{5}}{C_{4}R_{9}}=\tau_{0} \end{array}\right.$$

(16) $$\left\{\begin{array}{l} R_{1}=\frac{1}{a\tau_{0}C_{1}}\\ R_{2}=\frac{g_{1}}{\tau_{0}C_{1}}\\ R_{3}=\frac{1}{b\tau_{0}C_{2}}\\ R_{4}=\frac{g_{2}}{\tau_{0}C_{2}}\\ R_{5}=\frac{g_{3}}{\tau_{0}C_{3}}\\ R_{6}=\frac{g_{4}}{\tau_{0}C_{3}}\\ R_{7}=\frac{1}{\tau_{0}C_{3}}\\ R_{8}=\frac{1}{0.5\tau_{0}C_{4}}\\ R_{9}=\frac{g_{5}}{\tau_{0}C_{4}} \end{array}\right.$$

**Figure 22:**
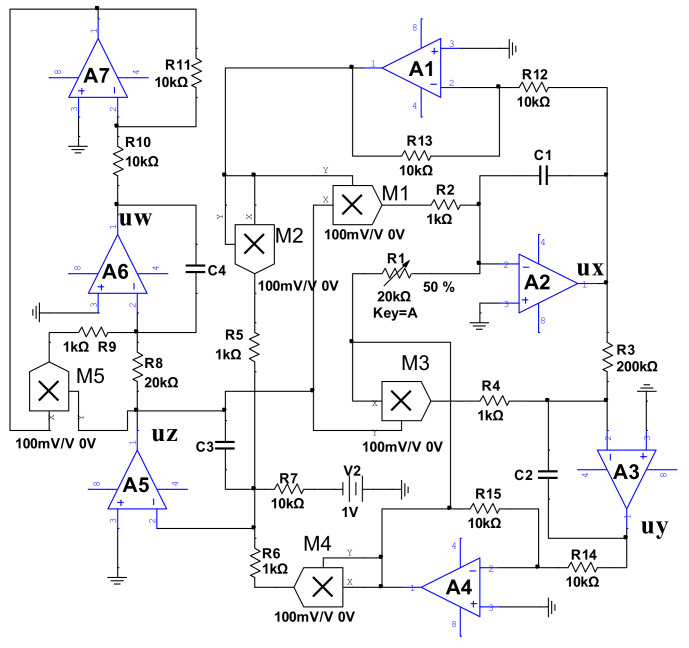
Circuit exhibiting hidden attractors without equilibrium.

In order to calculate and guarantee the unity of the circuit parameters, let the five multipliers and the four capacitors have equal gains, namely, $g_{i}=0.1V$(i=1,2,3,⋯5) and $C_{i}=10nF$(i=1,2,3,4). When b=0.05 is substituted into Equation (16), we can obtain $R_{1}=\frac{10}{a}k\Omega$, $R_{3}=\frac{10}{b}k\Omega =200k\Omega$, $R_{2}=R_{4}=R_{5}=R_{6}=R_{9}=1k\Omega$, $R_{7}=10k\Omega$, and $R_{8}=20k\Omega$. The circuit schematic, designed using Kirchhoff’s laws, is shown in Figure 22, where ux, uy, uz, and uw marked in the figure correspond to the output variables $u_{x}$, $u_{y}$, $u_{z}$, and $u_{w}$, respectively, and the resistance parameter of the inverter is $R_{10}=R_{11}=R_{12}=R_{13}=R_{14}=R_{15}=10k\Omega$. To observe the phase diagrams of circuits with hidden attractors, a sliding rheostat $R_{1}=20k\Omega$ was used (Figure 22).

### 5.2. Multisim Results

**Figure 23:**
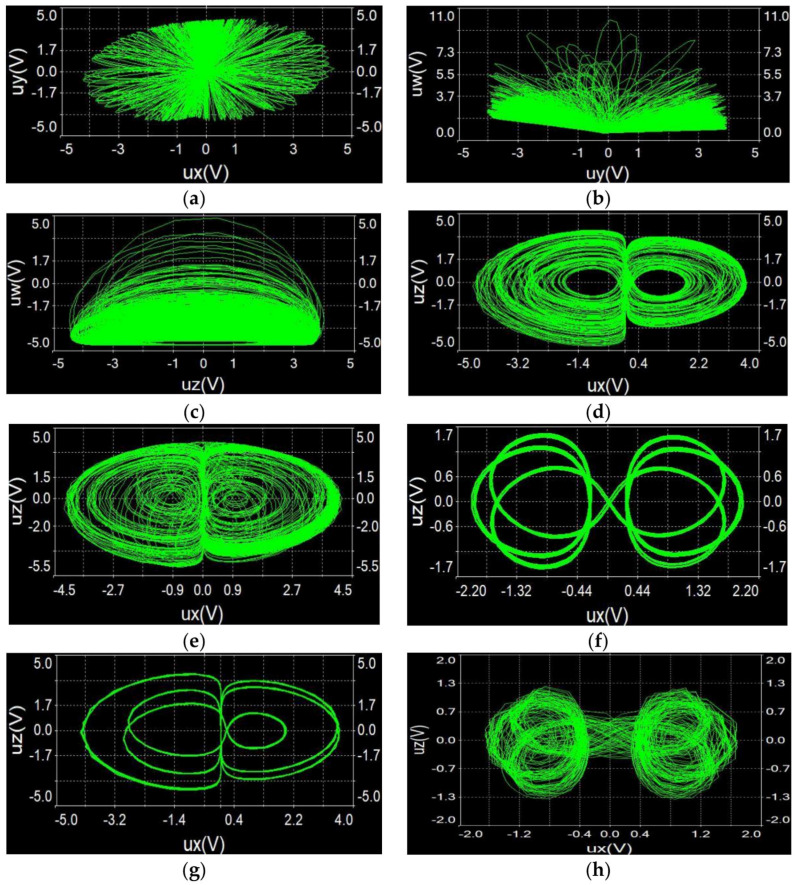
Hidden chaotic attractors of circuit (14) : (**a**) a=1, $R_{1}=10k\Omega$, $u_{x}-u_{y}$ plane; (**b**) a=1, $R_{1}=10k\Omega$, $u_{y}-u_{w}$ plane; (**c**) a=1, $R_{1}=10k\Omega$, $u_{z}-u_{w}$ plane; (**d**) a=4.9, $R_{1}=2.041k\Omega$, $u_{x}-u_{z}$ plane; (**e**) a=1.0621, $R_{1}=9.415k\Omega$, $u_{x}-u_{z}$ plane; (**f**) a=0.88922, $R_{1}=11.246k\Omega$, $u_{x}-u_{z}$ plane; (**g**) a=2.982, $R_{1}=3.353k\Omega$, $u_{x}-u_{z}$ plane; (h) a=1.2, $R_{1}=8.333k\Omega$, $u_{x}-u_{z}$ plane.

We used Multisim14.0 software to build the circuit shown in Figure 22. It is well known that the components used in Multisim software are highly compatible with the actual components. Therefore, the specific circuit scheme can well reflect actual circuit performance. Here, by adjusting the tap of the sliding rheostat to change the value of $R_{1}$, the projections of hidden attractors observed from the oscilloscope are displayed in Figure 23, which agree well with the phase diagrams of Equation (2) shown in Section 3.1. This also confirmed that the proposed system is physically achievable.

## 6. Discussion

The multisim results confirmed that the proposed hyperchaotic system with hidden attractors does not have transitional chaos or transient behavior. The slightly changing values of electronic components can greatly affect the state of a system with hidden attractors. Although the nonlinear characteristics of this system have been carefully studied, the impact of accuracy and simulation time on the system remains to be further studied. This aspect also requires scholars to do deep theoretical research work.

## 7. Conclusions

In this study, a chaotic mathematical model with hidden attractors was constructed. Firstly, system parameters and initial values were found to affect system dynamics and system complexity. From the analysis of the bifurcation characteristics of the system parameters, it was found that there were complex hidden dynamic behaviors, such as periodicity, quasi-periodic, chaotic, and hyperchaotic. In particular, under different initial conditions, different topologies of chaotic attractors or quasi-periodic limit cycles coexisted with chaotic attractors, and quasi-periodic limit cycles coexisted with chaotic attractors of various topologies. This shows that the proposed system has multistable characteristics. Moreover, the entropy of the system was analyzed from several aspects, such as the entropy of changing parameters, the entropy of different initial values, and the entropy of the chaotic characteristics of the system parameters, proving that the proposed system has very rich dynamic characteristics. Finally, the behaviors of hidden attractors were observed in electronic circuits by the method of improved modular design.

The numerical simulations and circuit implementation presented in this paper prove that the proposed system is a multistable system. Because it is very sensitive to the initial value and has a rich topological structure, the system is suitable for encryption applications.
